# Standards of resuscitation during inter-hospital transportation: the effects of structured team briefing or guideline review - A randomised, controlled simulation study of two micro-interventions

**DOI:** 10.1186/1757-7241-19-15

**Published:** 2011-03-03

**Authors:** Christian B Høyer, Erika F Christensen, Berit Eika

**Affiliations:** 1 Centre for Medical Education, Faculty of Health Sciences, University of Aarhus, Denmark; 2 Central Region Denmark, Department of Prehospital Medical Services, Denmark

## Abstract

**Background:**

Junior physicians are sometimes sent in ambulances with critically ill patients who require urgent transfer to another hospital. Unfamiliar surroundings and personnel, time pressure, and lack of experience may imply a risk of insufficient treatment during transportation as this can cause the physician to loose the expected overview of the situation. While health care professionals are expected to follow complex algorithms when resuscitating, stress can compromise both solo-performance and teamwork.

**Aim:**

To examine whether inter-hospital resuscitation improved with a structured team briefing between physician and ambulance crew in preparation for transfer vs. review of resuscitation guidelines. The effect parameters were physician team leadership (requesting help, delegating tasks), time to resuscitation key elements (chest compressions, defibrillation, ventilations, medication, or a combination of these termed "the first meaningful action"), and hands-off ratio.

**Methods:**

Participants: 46 physicians graduated within 5 years. Design: A simulation intervention study with a control group and two interventions (structured team briefing or review of guidelines). Scenario: Cardiac arrest during simulated inter-hospital transfer.

**Results:**

Forty-six candidates participated: 16 (control), 13 (review), and 17 (team briefing). Reviewing guidelines delayed requesting help to 162 seconds, compared to 21 seconds in control and team briefing groups (p = 0.021). Help was not requested in 15% of cases; never requesting help was associated with an increased hands-off ratio, from 39% if the driver's assistance was requested to 54% if not (p < 0.01). No statistically significant differences were found between groups regarding time to first chest compression, defibrillation, ventilation, drug administration, or the combined "time to first meaningful action".

**Conclusion:**

Neither review nor team briefing improved the time to resuscitation key elements. Review led to an eight-fold increase in the delay to requesting help. The association between never requesting help and an increased hands-off ratio underpins the importance of prioritising available resources. Other medical and non-medical domains have benefited from the use of guidelines reviews and structured team briefings. Reviewing guidelines may compromise the ability to focus on aspects such as team leading and delegating tasks and warrants the need for further studies focusing on how to avoid this cognitive impairment.

## Introduction

Urgent inter-hospital transfer poses a risk of making things worse to the patient: urgent transfer from secondary hospitals to tertiary hospitals is associated with increased mortality and morbidity [1-5].

Junior physicians are often expected to manage urgent inter-hospital transfer of critically ill patients needing higher levels of diagnostic or therapeutic interventions, e.g. percutaneous coronary intervention (PCI). The patient's health may be endangered by the consequences of, e.g., staff working under time pressure, unfamiliar surroundings, unusual equipment, unaccustomed co-workers or unfamiliar shifts in responsibilities and roles, all potentially leading to insufficient preparation or use of equipment, or sub-optimal diagnosis or treatment [1,2,4-7]. A common denominator underlying these examples is, that they can be contributed either to insufficient training or to lack of experience [3].

### Information overload

Maintaining focus in complex situations, such as during resuscitation, is challenged by the flood of clinical information to be collected, analysed, prioritised, and responded to (i.e. changes in the patient's colour or breathing pattern, the relevance of equipment alarms, and the importance of feedback from other team members). This flood of information may inundate the physician and cause a state of *information overload *which in turn may reduce cognitive skills, impair decision making, and decrease performance [8-11].

### Resuscitation guidelines as a filtered source of knowledge

One way to counteract information overload is to use filtered sources such as guidelines. Resuscitation guidelines constitute an organised summary of accumulated expert-level knowledge in the field [12]. Probably on this foundation, the mantra throughout resuscitation education has for many years been guidelines, guidelines, guidelines. However, as resuscitation is not only a matter of technical skills, like compression depth and frequency, it is also necessary to look at resuscitation as a whole. In order to identify weak spots in the care of acutely ill patients, it is necessary to apply an integrated approach considering not only the role of guidelines, but also factors as surroundings, equipment, and team work.

### Team work and team briefing

Team work is essential in resuscitation, as it combines professionals' skills and knowledge both within and between specialties [13,14]. A strategy to improve this intra- and inter-disciplinary cooperation is the use of team briefings, that is, a face-to-face meeting that ensures relevant information is shared within the team [4,13-15]. The benefit of team briefings, often supported by the use of checklists, has been established in the literature, e.g. by lowering the incidence of wrong side surgery or improving the overall performance [4,15-18].

In a previous simulation study on junior physician skills and behaviour in resuscitation,[5] we assessed the technical quality of resuscitation in a scenario with a simulated cardiac arrest victim. The scenario was designed as an inter-hospital transfer of a simulated patient with acute coronary syndrome (ACS) who developed cardiac arrest during the transfer. A total of 72 junior physicians participated in the simulations that were performed in genuine ambulances with ambulance crew as team members. We found junior physicians' were deficient in team leader skills, especially in terms of delegation of manual tasks. Another finding was wide variations in key-elements in resuscitation, such as time to first chest compression and first defibrillation. However, it could not be determined whether this was caused by insufficient knowledge and skills in resuscitation guidelines or by deficient team leader skills.

Knowledge about guidelines is without doubt crucial, but also team briefings, often structured by checklists, has successfully improved performance and reduced errors in medical practice [15-18]. Therefore, we want to test whether the standard of resuscitation is improved by review of guidelines *or *by a checklist based structured briefing, both performed just prior to the simulation.

### Aim

This study examines if introduction of one of two clinically applicable micro-interventions (review of guidelines or structured team briefing) affect the standard of team leadership and resuscitation in a simulated cardiac arrest scenario (during inter-hospital transfer of a simulated patient with ACS) by comparison of three groups (control and two intervention groups).

## Methods

The study design was an observational simulation study including three groups: a control group, a review group, and a team briefing group.

### Participants, recruitment, and ethics

Eligible participants were physicians who graduated within the last five years at time of the study, and functioned as 'front-line personnel' in Internal Medicine departments with acute admissions in 'Central Region Denmark'. The participants were recruited via consultants responsible for educating junior physicians in each department, and simulations were held during work hours on dates with the largest possible number of participants available. Under these circumstances, 46 physicians were able to participate. The participation was voluntary and data kept anonymous and confidential. Neither The Central Denmark Region Committees on Biomedical Research Ethics nor the Danish Data Protection Agency stipulated approval for this study. Although not legally necessary, we obtained informed consent from the participants in order to adhere to the higher standards for biomedical research.

### Equipment, set-up, and scenario

The simulation was held in a genuine and fully operational ambulance with usual personnel and standard equipment. The crew (a paramedic and an emergency medical technician (EMT)) were qualified in BLS and defibrillation, among other things, but not in administration of intra-venous drugs. During the entire simulation, the paramedic stayed in the patient's compartment. The EMT acted as the driver when the simulation was initiated, but the physician could request his assistance in the patient's compartment at any time. The facilitator (the first author of this paper) initiated the simulation by verbally announcing that the ambulance was now on the road and approximately halfway between the two hospitals. A manikin was placed supine on the stretcher, and the cardiac rhythm, peripheral blood saturation, and blood pressure were simulated via computer control [19,20]. Prior to the simulation, intravenous accesses were established, supplemental oxygen provided, and self-adhesive defibrillation pads attached.

The case was standardised and involved a patient with ACS requiring transfer to a tertiary hospital for percutaneous coronary intervention (PCI). During the transfer, the simulated patient developed a ventricular fibrillation (VF) cardiac arrest which was refractory to treatment for five minutes (Table 1). For the following three minutes delivery of DC-shock would induce return of spontaneous circulation (ROSC), which otherwise would happen spontaneously no later than eight minutes after the onset of VF in order not to burden the participant emotionally. The ambulance crew was instructed to be helpful but leave the task assignment and treatment decisions to the physician.

**Table 1:** Timeline of experimental protocol

	Events in the ambulance
"Departure" (T_-3 min_)	Initiation of the simulation: Facilitator announces: "the ambulance is on the road -has been driving for app. 30 minutes." Patient awake and stable, sinus rhythm and SpO_2 _99% on monitor. Engages in a conversation with the physician to attract the physician's focus. If physician stays focused on the monitor/defibrillator, conversation-time is prolonged.
	
T_-0:15 min_	Patient develops VT, pulse rate 180, blood pressure 80/50 mmHg. Complains about nausea and dizziness.
	
T_0 min_	Rhythm changes to VF. Unresponsive, vital signs absent VF is intractable in the following 5 minutes, regardless of the treatment given.
	
T_5 min_	VF changes to pVT sensitive to defibrillation. pVT converts to SR if defibrillated EMT offers help twice - enters only the patients' compartment if accepted by the physician.
	
T_8 min_	Sinus rhythm reappears, regardless of the treatment given.
	
T_end_	End of simulation. The patient is now somewhat responsive.

T_0 _indicates the time of onset of ventricular fibrillation, and _the subscript _text describes the timing of events before and after T_0_. Although parked during the simulation, it was clearly stated when the ambulance was "on the road." Abbreviations: SpO_2_: peripheral blood saturation, VT: ventricular tachycardia, VF: ventricular fibrillation, pVT: pulseless ventricular tachycardia, SR: sinus rhythm, ECG: Electrocardiogram, EMT: Emergency Medical Technician.

### Randomisation

Randomisation was done on an individual basis by letting the physician draw a closed envelope prior to the simulation. The envelopes included one of the numbers 1, 2, or 3, and were made, as well as shuffled, by an independent person prior to the entire study sequence. The interventions could not, by their nature, be blinded to the participant, the ambulance crew, or the investigator who conducted the simulation. However, as the participants did not know the nature of the interventions, they were not able to discern to which group they were randomised.

### Control group (n = 16)

Physicians randomised to the control group were given a short, written outline of the case and a verbal outline of the features of the manikin. The physicians randomised to the two intervention groups were introduced similarly.

### Team briefing group (n = 17)

**Figure 1:**
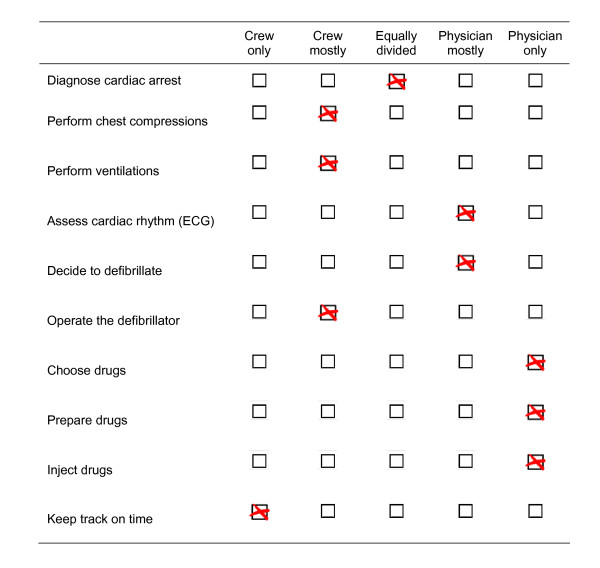
**Team briefing checklist.**  Items used in the team briefing checklist. To guide the discussion, each item was followed by five possibilities: 1) crew only, 2) crew mostly, 3) evenly divided, 4) physician mostly, and 5) Physician only. The optimum allocation of tasks was based on qualifications, competencies, and experience (for example, the ambulance crew was not qualified to administer intravenous injections). The optimum allocation, in the authors' opinion, is marked for each item (X). ECG: electro-cardio-gram

Physicians in the team briefing group were given a checklist reflecting the headlines in the resuscitation algorithm (Figure 1). The checklist was developed by the authors and items primarily based on our observations from our first study [5]. The physicians were instructed to utilize the time they felt was necessary to read the checklist and to brief the ambulance crew. To guide the discussion, each item was followed by five possibilities: 1) ambulance crew only, 2) mostly ambulance crew, 3) evenly distributed, 4) mostly physician, and 5) physician only. The optimum allocation of tasks, as illustrated in Figure 1, was defined on a consensus decision between the authors, based on formal information about the general qualifications and competencies held by the physicians and the ambulance crew, respectively (for example, the ambulance crew were not qualified to do intravenous injections).

### Review group (n = 13)

**Figure 2:**
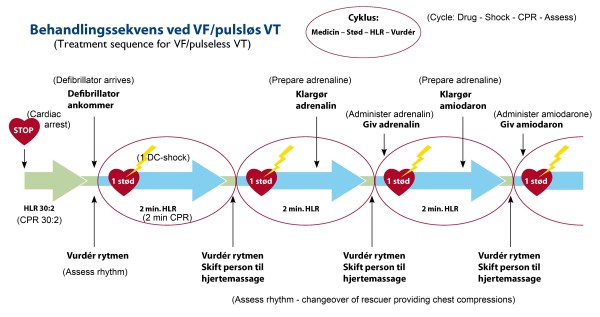
**Danish ALS-guidelines folder.** The Danish version of ALS guidelines handed out to the participants in the "review" group. The figure shows one page of the folder's four pages. English translations of the Danish text are given in (parentheses).

The physicians in the guideline-review group were given the current resuscitation guidelines published by the Danish Resuscitation Council (DRC) and were told to use the time they felt was necessary to study the guidelines folder prior to initiation of the simulation [21]. It should be noted, that the participants did not receive any education or training in the guidelines in connection to the study. The guidelines folder (Figure 2) was the official DRC translation to Danish of the English version of the guidelines published by the European Resuscitation Council in 2005 [22].

### Data collection, indicators, and analysis

Prior to participation, a questionnaire was filled in including items about age, gender, date of birth, date and place of graduation, and participation in pre- and postgraduate resuscitation courses. All simulations, including interventions, briefings, and debriefings were recorded on video, and data was subsequently extracted by manual review of the recordings. The time allocated to reviewing guidelines or conducting team briefing was recorded, as was the time for chest compressions, defibrillations, ventilations, administration of pharmacological agents, and time elapsed until the physician requested assistance from the driver. Hands-off ratio was defined as the total time without chest compressions divided by the total time with a non-perfusing cardiac rhythm (in this case the time from debut of VF to ROSC).

### Effect parameters

The following effect parameters were used to compare the three groups:

The evaluation of the junior physicians team leadership in relation to the ambulance crew was based on whether the physician a) requested help, and in case this happened, when it was done, and b) delegated tasks, and, in case of this, which tasks. Delegated tasks were defined as any task the physician asked the ambulance crew to perform, whether or not they would have initiated these by themselves in a real situation (chest compressions, ventilations, or alike). The evaluation of the resuscitation standard was made by measurement of the elapsed time from debut of ventricular fibrillation to the initiation of a) defibrillation, b) chest compression, c) ventilation, d) pharmacologic treatment, and e) hands-off ratio (the time without ongoing chest compression over the time in cardiac arrest).

To adjust for the somewhat competing nature of the actions a)-d) mentioned above, the actions were collated in one variable describing the "first meaningful action", that is, the time elapsed from the debut of ventricular fibrillation to the initiation of any of those (defibrillation, chest compression, ventilation, or pharmacologic treatment).

### Statistics

Video recordings from a digital surveillance camera mounted in the ambulance documented all simulations, and recordings were continuously time-stamped by the camera with a built-in on-screen digital clock. The data were entered in an Access 2003 database[23] and statistical analysis performed with Stata/IC 10.1 [24]. Nominal data were expected to have a non-Gaussian distribution and wide variations, so the Kruskal-Wallis equality-of-populations rank-test was used [5]. The data are presented as median (range) [1^st^; 3^rd ^quartiles]. Pearson's χ^2^-test was used on categorical data. Three simulations were randomly selected and used for calculation of intra- and inter-observer variability. All simulations were reviewed twice by two independent persons (a physician and a medical student), as well as by the first author of this paper. The inter- and intra-observer correlation coefficients were calculated using Stata/IC 10.1 [24].

## Results

### Participants

**Figure 3:**
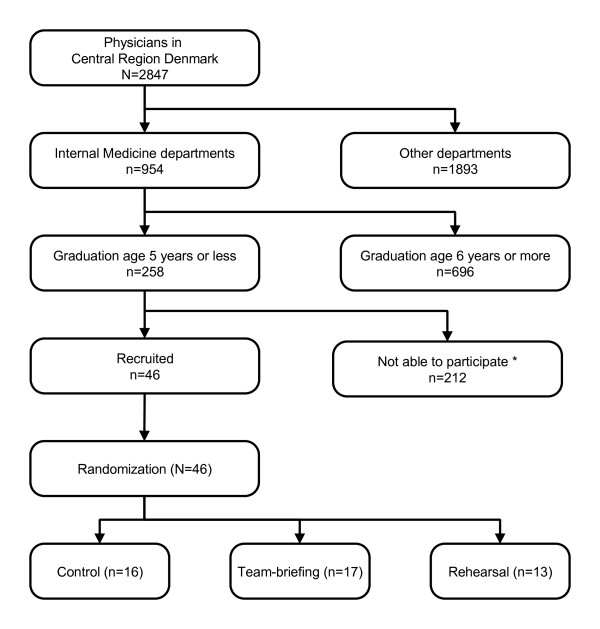
**Participants.** Modified Consort-flowchart diagram showing the entire population from which the participants were chosen. A total of 258 physicians were eligible for enrolment in the study, of which 46 (18%) participated. Reasons why eligible physicians could not participate were numerous; e.g. participants who had already volunteered to participate were hindered in participation due to extreme workload, changes in work-schedule, exhaustion after night-duty, or alike.

A modified Consort flowchart is shown in Figure 3. Eligible for the study were 258 physicians; 46 were recruited. Median age of participants was 29 years (27-44) [27; 31), sex ratio was 1:1 (23 males, 23 females) and average graduation age 1 year. No statistically significant differences between the groups were identified. The median time used were 104 seconds (51-201) [57; 144] for reviewing resuscitation guidelines and 203 seconds (111-329) [157; 293] for conducting a team briefing.

### Team leadership in relation to ambulance personnel: requesting help

**Figure 4:**
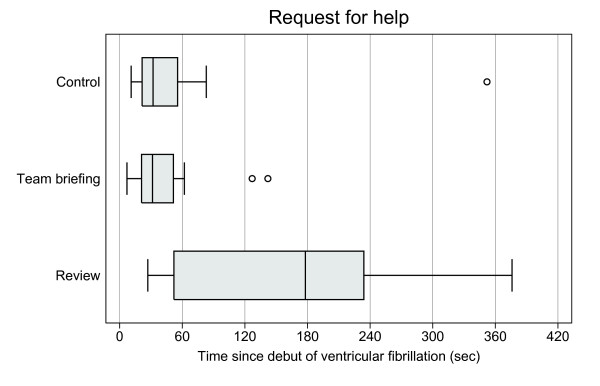
**Time for request for help since debut of ventricular fibrillation.** Time from debut of ventricular fibrillation cardiac arrest to physicians' calling for the driver's help in the patient's cabin (n=39). The distribution among the three groups was significantly different (Kruskal-Wallis, p = 0.021). Boxes are median and upper/lower quartiles, whiskers upper/lower adjacent values, and dots outliers (one extreme outlier is not depicted in the control group).

In the review group, help was requested later than in the other groups: median time was 162 seconds compared to 21 seconds in both the control and the team briefing groups (Kruskal-Wallis, p = 0.015, Table 2, Figure 4). The physicians did not request the driver's help at all in 15% of cases (7/46): control group 19% (3/16), review group 23% (3/13), and team briefing group 6% (1/17), with no statistical differences between groups. Never calling for help was associated with a statistically significant increase in hands-off ratio (Kruskal-Wallis, p = 0.016). The median hands-off ratio if help was not requested was 54%, compared to 39% if the driver's assistance in the patient's compartment was requested (p < 0.01).

**Table 2:** Time for key resuscitation procedures since debut of ventricular fibrillation

	Minimum	1^st ^quartile	Median	4^th ^quartile	Maximum
Time to request for help *					

Control (n = 13)	1	15	21	49	530
Team briefing (n = 16)	1	5	21	39	111
Review (n = 10)	14	41	162	224	364

Time to first chest compression					

Control	17	26	36	55	276
Team briefing	8	22	38	51	86
Review	15	31	40	51	71

Time to first defibrillation					

Control	17	31	64	75	168
Team briefing	18	40	63	84	322
Review	18	38	58	90	213

Time to first ventilation					

Control	30	40	53	79	114
Team briefing	37	56	64	81	128
Review	36	49	62	79	130

Time to first pharmacologic treatment					

Control	34	210	232	257	365
Team briefing	102	227	291	365	399
Review	142	173	261	331	437

Time to first meaningful action					

Control	17	19	27	35	74
Team briefing	8	22	36	46	57
Review	15	26	37	43	60

Time to first request for help, first defibrillation, first chest compression, first ventilation, and first pharmacologic treatment in the three groups: control, team briefing, and review of advanced life support guidelines.

### Team leadership in relation to ambulance personnel: delegating tasks

Chest compressions were delegated entirely to the ambulance crew in 63% (10/16) and 65% (11/17) of cases in control and team briefing groups, respectively, but only 31% (4/13) in the review group. Defibrillation was delegated entirely to the ambulance crew in 62% (6/16), 53% (9/17), and 15% (2/13) and ventilations in 69% (11/16), 82% (14/17), and 69% (9/13), in the control, team briefing, and review groups, respectively. Statistically significant differences were not found. Drug administration was never delegated to the ambulance crew.

### Evaluation of the resuscitation standard: time to initiation of procedures

**Figure 5:**
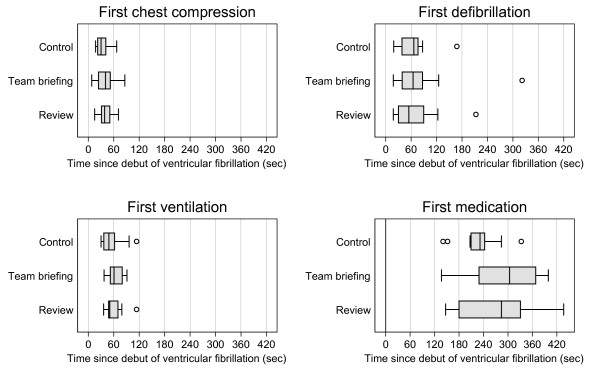
**Time for other key resuscitation procedures since debut of ventricular fibrillation.** Time from debut of ventricular fibrillation cardiac arrest to find chest compression, first defibrillation, first ventilation, and first drug administration. Boxes are median and upper/lower quartiles, whiskers upper/lower adjacent values, and dots outliers.

**Figure 6:**
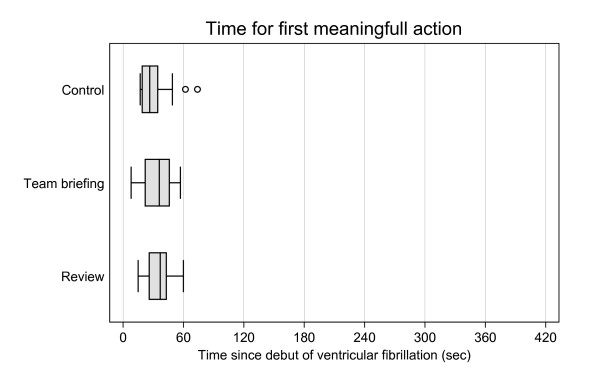
**Time for first meaningful action since debut of ventricular fibrillation.** Time from debut of ventricular fibrillation cardiac arrest to first meaningful action, that is, chest compression, first defibrillation, first ventilation, or and first drug administration. Boxes are median and upper/lower quartiles, whiskers upper/lower adjacent values, and dots outliers.

Comparisons between control, review, and team briefing groups in terms of time to first chest compression, defibrillation, ventilation, and pharmacologic treatment revealed no significant differences (Table 2, Figure 5). The time elapsed from debut of the cardiac arrest to the first meaningful action (chest compression, defibrillation, ventilation, or pharmacologic treatment) showed no statistically significant differences (Table 2, Figure 6).

### Evaluation of the resuscitation standard: hands-off ratio

The hands-off ratio did not differ significantly between the three groups; in the control group it was 42% (21-86) [36; 50], in the review group 44% (34-59) [38; 50], and in the team briefing group 37% (24-60) [34; 44] (Kruskal-Wallis, p = 0.27).

The intra-observer variability coefficients were 0.9966, 0.9981, and 0.9971, respectively, and the inter-observer variability coefficients were 0.9897, 0.9913, and 0.9906, respectively.

## Discussion

An unexpected finding in our study was that the review of guidelines prior to the simulation test was associated with a statistically significant, almost eight-fold, increase in the delay in requesting the EMT to help in the patient's compartment.

This finding suggests a possible 'inhibitory' effect of reviewing guidelines on resource thinking. A possible explanation is that focusing the working memory on technical guidelines impairs the physicians' overall perspective, as working memory has a limited capacity. The limited capacity may again contribute to information overload and consequently compromise the overview.

It has been documented, that lack of overview jeopardize teamwork and consequently decrease the standard of the resuscitation [4,12,16,25-30].

Although not statistically significant, a similar pattern of lowest delegation rate in the review group than in the other two groups was seen in delegation of chest compressions and defibrillation.

### Hands-off ratio

The incidence of successful resuscitation, in real life, increases significantly with the performance of on-going chest compressions [31-35]. Therefore, the hands-off ratio may be the most important parameter in the evaluation of resuscitation tests [36]. Never calling for help from the driver was associated with a statistically significant increase in hands-off ratio, rising from 39% if help was requested to 54% if not (p < 0.01). This finding suggests that stopping the ambulance and unifying all available human resources in the patient's compartment may be better than keeping the ambulance moving, thereby leaving the EMT in the driver's cabin.

### Strengths and limitations

This simulation study was performed in an authentic setting, as a real ambulance was used and the EMTs on duty on the day of the experiment comprised the team together with the junior physician studied. By choosing this model, it became possible to study the isolated performance of a junior physician working in a small resuscitation team as opposed to a larger, in-hospital team, with senior physicians taking the lead.

The small-intervention format was chosen because the interventions should be easy to administer, should cause no delay in resuscitation attempts, and, in case of success, be easy to implement. The interventions were tested on junior physicians because we had previously shown how this group of physicians showed large variations in their resuscitation skills [5].

While simulation offers many advantages, it also has potential problems. First, it is impossible to keep all variables completely stable, as different EMTs and ambulances were used according to time and place for the simulations. Second, the set-up increases the complexities of the tasks facing the junior physician because he or she has to deal with a number of unfamiliar challenges: should medicine be prepared in syringes in advance, where to put the medicine bag, and where to place oneself, just to mention a few choices that must be made by the junior physician. These drawbacks and potential sources of bias of our "mobile laboratory" are, however, worthwhile in our opinion, as the setting allows us to get a better understanding of the real performance of young physicians compared to a conventional laboratory setting.

Participants were all volunteers. This could introduce bias if only physicians proficient in ALS - or feeling so, at least - would participate, perhaps after refreshing guidelines. If this was the case, the results may be even more relevant as these would then reflect the better part of the group and thereby overestimate the skills in the underlying population. Regarding the number of participants, an unfortunate, but frequent, problem was, that participants who had already volunteered to participate were hindered in participation due to extreme workload, changes in work-schedule, exhaustion after night-duty, or alike. This caused the number of participants to be lower than we expected from our previous experiences [5].

Comparisons of the three groups (control, team briefing, and review) showed no differences when it came to technical resuscitation skills. This could be because the interventions were too small-scaled to trigger results. However, it is also possible that the lack of statistically significant effects is due to the possibility of a type 2 error (rejecting an effect that is there) due to small sample sizes (13, 16, and 17 participants in the groups, respectively). Another explanation could be the possibility of a ceiling effect, that is, if the participants had high levels of resuscitation skills before the experiment it would leave little room for improvement. Also, the intra-group variation was large and might have outweighed the inter-group variation and thereby masked possible differences. Finally, imprecise data collection could also be an explanation for the large variations, however, as the intra- and inter-observer variability coefficients were all very high (> 0.98) this is unlikely.

### Perspectives

*Fixation errors *are known from clinical practise: information that should change the course of the resuscitation attempt is ignored (the team leader believes cardiac arrest is due to myocardial infarction and ignores intelligence about ingestion of toxic substances, e.g.) [37]. The underlying mechanism may be *inattentional blindness*, a term from the psychological literature, describing how a preoccupied mind may fail to shift attention intentionally [38]. Rehearsing guidelines may cause the physician to focus strictly on following guidelines and thereby miss being a team leader. A parallel to this can be the first phase of learning new skills: focus of the novice will be to understand the task and avoid mistakes [39].

A team briefing may help the junior physician to delegate tasks, as it ensures that relevant information is shared between the team members and helps the team to agree on goals, identify key priorities, and identify ways to reach these goals [4,13-15,40]. It may be especially important to help junior physicians to value teamwork, as they often feel time-pressured and therefore may be reluctant to invest time in team briefings [41].

Hunt et al. have advocated for team briefing prior to hospital transfer of critically ill patients [4]. In a recent study, Westli et al. investigated teamwork skills and behaviour correlated to medical treatment quality of trauma teams in simulated settings. They suggested that trauma teams could be significantly more effective if communication and information exchange skills were strengthened by team briefing and establishing a shared mental model [42]. This is further emphasized by Neily et al. who recently published how a medical team training program reduced surgical mortality during a three-year period with more than 180,000 procedures examined [43]. Hunziker et al. compared the influence of a short leadership instruction versus a short technical instruction in a high-fidelity cardiac arrest simulation [36]. Among their findings was, that leadership instruction resulted in a better overall performance regarding time to first compression and hands-off time, while technical instructions, although improving e.g. arm position during chest compressions, did not. Our findings seem to be coherent with Hunziker et al. as we found review of guidelines delayed requesting help

The use of checklists and timeouts has been shown both to improve understanding and to mitigate potential errors, especially in elective surgery [15-18]. Our study suggests that similar advantages can be made with the transportation of unstable patients.

Rall et al. has proposed the "10-seconds-for-10-minutes-principle", a metaphor for a formal "time out" during teamwork. They argue, that time "lost" by a quick team briefing (the "10 seconds") is "won" by a massive increase in team efficiency that saves time, improve treatment, and increases safety (the "10 minutes") [41]. Translated to practise, they teach the team leader to take the time needed, take a deep breath instead of making a quick diagnosis and start treatment within a second, but make a formal time out instead [41].

Our structured team briefing intervention took place under relaxed circumstances, and the physicians were told to use the time they felt necessary resulting in a mean time consumption of 206 seconds (approximately 3 1/4 minutes). Our data do not allow us to extrapolate to time consumption in real clinical life. It does however equal findings from Lingard et al. in an evaluation of a preoperative checklist where 92% of team briefings took between 1-4 minutes [16]. Thus it seems plausible that a structured team briefing could be applied at real inter-hospital transportations without consuming too much of precious time.

Our results suggest that reviewing guidelines might compromise the ability to focus on other aspects of resuscitation, such as teamwork, warrants the need for further studies focusing on how to avoid this cognitive impairment. However, our recommendation goes further than just a proposal for conducting additional studies: we believe it is necessary to perform this under very realistic settings, or, as Yeung and Perkins has stated, to do 'road testing of how treatment algorithms derived from evaluation of resuscitation science interact with each other before their application in real life.'[44]

## Competing interests

The authors declare that they have no competing interests.

## Authors' contributions

Authors CBH, EFC, and BE have all contributed to the conception and design of the study, to the interpretation of data, drafting and revision the manuscript and approved the final version to be published. Further, author CBH has contributed to the acquisition of data.
